# Clinical Scenarios Emerging from Combined Immunophenotypic, Molecular and Morphologic Analysis of Pancreatic Cancer: The Good, the Bad and the Ugly Scenario

**DOI:** 10.3390/cancers11070968

**Published:** 2019-07-10

**Authors:** Eva Karamitopoulou, Beat Gloor

**Affiliations:** 1 Pancreatic Cancer Research Group, Institute of Pathology, University of Bern, Murtenstrasse 31, CH-3008 Bern, Switzerland; 2 Department of Visceral Surgery, Insel University Hospital, University of Bern, Freiburgstrasse 18, CH-3010 Bern, Switzerland

**Keywords:** pancreatic cancer, immune phenotypes: molecular subtypes

## Abstract

Pancreatic ductal adenocarcinoma (PDAC) is a devastating disease with increasing incidence and dismal prognosis. The composition of the immune cell infiltrates in the tumor microenvironment (TME) and the dynamic interplay between cancer- and immune cells can influence and/or be influenced by tumor-intrinsic characteristics like molecular profiles and tumor cell morphology. The combined analyses of pancreatic cancer by using morphologic, genetic, and immunologic features help us understand the significant heterogeneity of the TME and recognize the different mechanisms of immune evasion. Moreover, this information may lead to the identification of novel biomarkers for more precise patient stratification and therapy guidance.

## 1. Introduction

Pancreatic ductal adenocarcinoma (PDAC) is a highly lethal neoplasm with rising incidence; it is predicted to become the second leading cause of cancer-related death in the next decade [1]. Despite recent improvements by using adjuvant chemotherapy with FOLFIRINOX [2] and latest developments regarding molecular subtyping, PDAC remains mostly resistant to known therapeutic modalities [3]. The use of immunotherapy, although beneficial in many solid tumors, has shown limited success in patients with pancreatic cancer [4]. So far, the PDAC patient subset that appears to respond to immunotherapy represents <2% of all PDAC patients, mainly restricted to tumors with microsatellite instability (MSI) [5]. Thus, many immunotherapy approaches that have been successful in other cancer types did not yield the expected results in PDAC [6]. These include IL-2, oncolytic viruses, checkpoint blockade, Transforming Growth Factor beta (TGFβ) inhibitors, neoantigen vaccines, Treg depletion, and CD47 blockade [7]. Although efforts can be made to incorporate these agents into combination therapies, their lack of efficacy as monotherapies suggests that there are several immunologic barriers that segregate together to help evade immuno-surveillance in PDAC. This can be mostly attributed to the complex and frequently immunosuppressive microenvironment of PDAC which is known to display low immunogenicity [8]. In this respect, recent evidence suggests a correlation between patient outcome and the composition, spatial organization, quantity and quality of tumor-infiltrating immune cells, which can influence the adaptive immune response in PDAC [9].

This review focuses on the significance of the different clinical scenarios that emerge after combined analysis of the immunophenotypic, morphologic and genetic features of the tumors, especially regarding the potential identification of novel strategies for a more targeted and individualized therapeutic approach to overcome immunosuppression and augment immunogenicity in the Tumor Microenvironment (TME) of PDAC.

## 2. Molecular Classification of Pancreatic Cancer

Although recent molecular classifications of PDAC based on sequencing studies have helped to stratify patients into molecular subgroups, their clinical value is still limited. Apart from a small number of rare targetable changes, such as microsatellite instability, *BRCA2* mutations and the less common *KRASG12C* mutations [10,11,12], the main driver mutations (*KRAS*, *TP53*, *CDKN2A* and *SMAD4*), known to occur in different combinations in most PDACs, or any other mutations including *KDM6A, RBM10* and *MLL3*, reported to occur at lower frequency, are not targetable [13]. Regrettably, there is currently no targeted therapy for the Rat sarcoma (RAS) pathway, which is the most important molecular driver of pancreatic tumorigenesis, since *KRAS* is mutated in more than 90% of PDACs and even most of the *KRAS* wild type PDACs display alterations that activate the RAS-Mitogen-Activated Protein Kinase (MAPK) pathway upstream or downstream of *KRAS* [11]. The accumulation of driver mutations seems however, to be of prognostic significance, as shown more recently by Qian and colleagues [14]. These authors, after analyzing protein expression and DNA alterations for the main PDAC drivers *KRAS, CDKN2A, SMAD4*, and *TP53* in 365 resected PDACs, reported that patients with *KRAS* mutations had worse disease free survival compared with patients with *KRAS* wild-type tumors and that PDAC-patients with a greater number of altered driver genes had worse disease free- and overall survival [14].

Collisson and colleagues [15], after analyzing expression data from human and mouse cell lines reported three prognostic molecular subtypes of pancreatic cancer: the classical, the quasi-mesenchymal and the exocrine-like. The classical subtype, which was the one with the best outcome, was characterized by a high expression of genes coding for adhesion specific molecules and epithelial differentiation. On the other hand, the quasi-mesenchymal subtype, which was associated with the poorest prognosis, showed higher expression of genes coding for mesenchymal differentiation. The exocrine subtype was reported to be associated with the expression of genes coding for digestive enzymes [15]. A few years later, Moffitt and colleagues [16], after incorporating data from primary and metastatic tumors as well as normal samples, identified specific gene expression patterns which segregated into two main pancreatic cancer subtypes: the classical, corresponding to the classical subgroup from the Collisson study [15] and exhibiting better outcome, and the basal, a poor prognostic subtype which mostly overlapped with the Collisson’s quasi-mesenchymal subgroup [15,16]. Lately, Bailey and coworkers [13], after genomic analysis of 456 PDACs identified 32 recurrently mutated genes aggregating into 10 pathways: Kirsten rat sarcoma (KRAS), TGF-β, Wingless-related integration site (WNT), NOTCH, ROBO/SLIT signaling, G1/S transition, SWI-SNF, chromatin modification, DNA repair and RNA processing. Expression data processing leaded to the identification of four prognostic subtypes: squamous, pancreatic progenitor, immunogenic, and aberrantly differentiated endocrine exocrine (ADEX). The squamous subtype expressed gene programs involved in the regulation of inflammation, hypoxia response and TGF-beta signaling and showed upregulated expression of TP63ΔN and frequent TP53 mutations, along with activation of Epidermal Growth Factor (EGF) signaling [13]. This subtype overlaps with the quasi-mesenchymal subgroup described by Collisson [15], as well as the basal subtype described by Moffitt [16] and was associated with worse overall survival. In contrast, the pancreatic progenitor group overlaps with the classical subtypes in the Collisson and Moffit classifications and displays better prognosis [13].

In subsequent analyses, however, performed by The Cancer Genome Atlas pancreas cancer, project only two PDAC subtypes could be verified: the basal-like, which identifies PDACs with poor prognosis and is characterized by basal markers, and the classical subtype, which is characterized by differentiated ductal markers and identifies PDACs with better prognosis [11]. The ADEX and immunogenic subtypes were shown to have low neoplastic cellularity, implying that normal tissues may have contributed to their molecular signatures [11]. A later study by Maurer et al. [17], after deconvoluting the stromal signatures for tumor-specific gene expression confirmed the existence of the two major epithelial subtypes of PDAC, simultaneously recognizing two primary subtypes of associated stroma, thereby underlining the importance of the TME in PDAC progression.

Molecular subtyping is still not part of the routine clinical workup of PDAC patients and is currently not widely used to inform treatment options. Nevertheless, opportunities are emerging that may lead to the identification of novel therapeutic targets. For example, the combined genomic, transcriptomic, and therapeutic profiling of patient derived organoids could predict therapeutic responses to chemotherapy in the neo-adjuvant, adjuvant and advanced PDAC settings [18].

## 3. Morphology Matters: The Important Role of Tumor Budding

**Figure 1:**
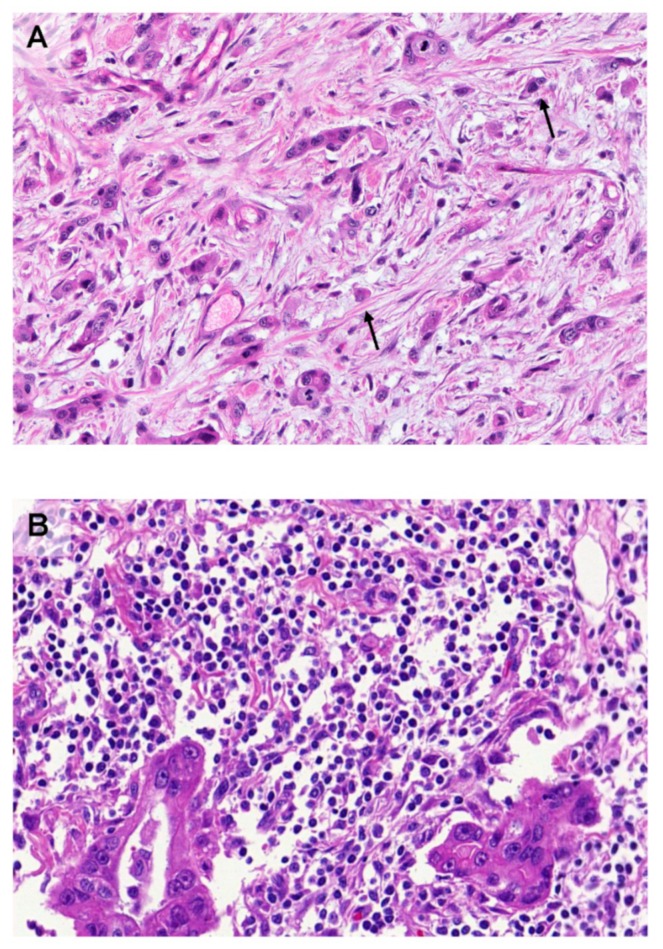
(**A**) PDAC with many tumor buds (arrows). The lack of immune cells in its microenvironment is evident. (**B**) PDAC with no tumor buds. Its microenvironment is particularly rich in immune cells. Hematoxylin-Eosin stained sections; ×200.

PDACs with more aggressive behavior are morphologically characterized by a large number of undifferentiated tumor cells, growing as single cells or small groups of up to four cells, disconnected from the main tumor, known as *tumor buds*, to be found both intratumorally and at the area of the invasive front [19,20]. Tumor budding has been repeatedly shown to be a strong and independent adverse prognostic factor in PDAC [19,20,21,22,23] (Figure 1). Furthermore, tumor budding has been reported to display properties of Epithelial Mesenchymal Transition (EMT) and budding cells are able to adopt a partial EMT state [24,25,26]. Indeed, tumor buds have been shown to have reduced E‑Cadherin expression, to lose membrane β‑catenin expression and to overexpress EMT associated biomarkers like zinc finger E‑box-binding homeobox 1 and 2 (ZEB1 and ZEB2), SNAIL and N-Cadherin [24,25,26,27]. Additionally, budding cells generally do not show apoptotic or proliferative activity, thus confirming that migration and proliferation cannot take place simultaneously [27].

MiRNA dysregulation in both tumor and stromal cells seems to also affect tumor budding [28]. Thus, tumor budding cells have been shown to have reduced expression of miR‑200b and miR‑200c, while on the other hand they exhibit increased expression of ZEB1 and ZEB2 [28,29]. The negative feedback between ZEB proteins and miRNA-200 family described in many carcinomas, is thought to act as a molecular regulator of the switch between the epithelial or the mesenchymal state of the cells during the process of EMT [30]. This mechanism seems to be employed by the tumor buds in order to achieve a partial EMT state. The contribution of surrounding stromal cells in this process, both by expressing high-levels of E-Cadherin suppressors and/or by enhancing the miRNA dysregulation, highlights the role of the stroma in establishing a microenvironment that is permissive to the development of tumor buds [24,28]. Since genetic alterations driving EMT features are an early phenomenon in tumorigenesis [27], all this data support that EMT phenotype can be intensified or attenuated by local factors in the TME opening opportunities for therapeutic intervention.

Interestingly, a number of findings associate EMT to the cancer stem cell phenotype [31], implying that tumor buds may represent a subpopulation of cancer stem cells. This is also supported by the fact that WNT, which is known to promote the stem cell-like phenotype [32], plays an important role also in the promotion of the tumor budding phenotype [24,33]. Moreover, EMT cells exhibit features analogous to cancer stem cells, for example they are drug resistant and are characterized by higher metastatic potential [34]. This may be one of the factors that explain the worse prognosis of PDACs exhibiting high-grade tumor budding.

## 4. Heterogeneity of Immune Cell Composition in the TME of Pancreatic Cancer

PDAC is considered to be an "immunosuppressive" neoplasm that can “utilize” several mechanisms of immune evasion. Although T-cells are abundant in the PDAC stroma, and patients with higher levels of CD4+ and/or CD8+ T-cells have been shown to have improved survival, most PDACs eventually develop an immunosuppressive microenvironment that hampers anti-tumor T-cell infiltration [35,36]. Common immune evasion mechanisms in this regard include the recruitment of regulatory immune cells, the secretion of immunosuppressive chemokines and cytokines as well as the expression of cell-surface proteins, like Programmed Death-Ligand 1 (PD-L1) and colony-stimulating factor 1 receptor (CSF1R) [37]. Thus, the different composition of the immune cell infiltrates in the TME could give us important clues regarding the different immunosuppressive mechanisms. For example, the immune microenvironment of a large number of PDACs shows increased infiltrates of T regulatory cells (Tregs) as well as myeloid-derived suppressive cells (MDSCs) mediated by the hypoxic conditions in the PDAC microenvironment, which can inhibit the anti-tumor activities of the effector T-cells [36,38,39]. Additionally, immunoediting can alter the immunogenicity of cancer cells steering the production of immune resistant clones [37]. 

A most interesting mechanism by means of which cancer cells can further promote immunosuppression is the upregulation of checkpoint inhibitor molecules like PD-L1 and cytotoxic T-lymphocyte-associated Protein 4 (CTLA4), which confer inhibitory signals to the immune cells [40]. PD-L1 has been shown to be upregulated in a subpopulation of PDACs conferring worse prognosis to the patients [35].

On the other hand, there seems also to exist a subpopulation of PDACs with a more cytotoxic and immunogenic microenvironment, characterized by abundant effector T-cells in association with reduced presence of immunosuppressive immune cells, a balance that confers a better outcome to the patients [36,39]. At the extreme end of this spectrum seem to be the rare mismatch repair (MMR) deficient PDAC cases with microsatellite instability (MSI-H) which exhibit a very immunogenic microenvironment with a high T-effector/T-regulatory cells ratio [39]. MMR deficiency is rare in PDAC, its frequency being reported at about 1%, with a prevalence in carcinomas arising from intraductal papillary mucinous precursors (IPMNs) [5]. The reported correlation of MSI-H tumors with clinical benefit after administration of immune checkpoint blockade therapy [41] renders testing for MMR deficiency in PDAC, especially in the metastatic setting, nonetheless inevitable.

Interestingly, it has been shown that not only the relative abundance but also the distribution of the T-cells and their spatial relationship with the cancer cells can give us further clues regarding their biologic interactions. Carstens and colleagues [36] showed that the anti-tumor effect of cytotoxic T-cells was positively correlated with their vicinity to the cancer cells.

Furthermore, the role of B-cells as a regulator of the immune response in the TME is starting to emerge. For example, the CD20+ and CD3+ stromal immune cell infiltrates can give rise to the formation of tertiary lymphoid tissue (TLT) which seems to convey a strong anti-tumor impact associated with survival advantage in PDAC [39,42].

Finally, there is contradicting evidence regarding the role of the stroma in the immune microenvironment of PDAC. Although stromal desmoplasia has long been hypothesized to hamper anti-tumor T-cell activity by preventing T-cells from attacking the tumor cells [43], more recent observations, have not confirmed an inhibitory role by the stroma since PDACs with differences concerning the abundance of pericellular cytotoxic T-cell infiltrates were not found to differ in the levels of aSMA and Collagen-I deposition [36].

PDAC, is a cancer driven mostly by recurrent gene copy number alterations than by recurrent mutations- indeed, three of the four driver mutations of PDAC, *TP53*, *CDKN2A* and *SMAD4,* affect tumor suppressor genes- and thus it has not been associated with increased neoantigen levels that could enhance immune infiltration [44,45,46]. Recently, Balachandran and colleagues [44] indicated that neoantigen quality, more likely than quantity, may influence immunogenicity in PDAC. These authors showed that specific neoantigens brought about during disease evolution, like neoantigens in mucin 16, evoke a strong cytotoxic immune response conferring survival benefit [44].

## 5. Clinical Scenarios

The presence of distinct PDAC-subtypes is supported by evidence from many studies showing that the significant variation concerning the genetic background and the immune cell composition of the microenvironment result to different phenotypic and prognostic/predictive categories [13,15,16,47]. Thus, it seems that various mechanisms can monitor the biologic interplay between the cancer- and the immune-cell populations, both at genetic and microenvironmental level, creating a spectrum of immunosuppressive conditions and leading to a great diversity in the nature of immune responses in the TME of PDAC. By combining the genetic, immunophenotypic and morphologic evidence distinct clinical scenarios emerge that may inform various prognostic/predictive PDAC subgroups.

The “good” clinical scenario comprises a minority of PDACs characterized by a cytotoxic immune phenotype. Their molecular and clinical features are more compatible with the pancreatic progenitor subtype by Bailey et al. [13] or the classical subgroup described by Collisson et al. [15] and Moffitt et al. [16]. These PDACs display an *“immune-rich”* microenvironment with abundant effector CD4+ and CD8+ T-cells, some of them with additional presence of TLTs, along with reduced presence of immunosuppressive immune cell populations. Morphologically, they are characterized by low-grade tumor budding and display favorable clinicopathologic features associated with prolonged survival [39,48].

The “bad” and unluckily more common clinical scenario comprises the majority of PDACs that display a TME rich in immunosuppressive populations and poor in effector T-cells indicating an *“immune escape”* mechanism for evading host immune response [49]. They are additionally characterized by a combination of genetic and microenvironmental factors that promote EMT, including somatic genetic alterations and miRNA dysregulation, giving rise to an aggressive phenotype with high-grade tumor budding, unfavorable clinicopathologic features and poor prognosis [39]. This supports the notion that tumor budding cells can interact with their microenvironment, helping to create those conditions that warrant their survival and further tumor growth. The molecular and clinical characteristics of this subtype largely overlap with the squamous subtype, as described by Bailey et al. [13], and/or the quasi-mesenchymal subtype described by Collisson et al. [15], and the “basal” subtype by Moffitt et al. [16]. In support of this, PDACs of the squamous subtype are characterized by gene programs and networks promoting EMT [13], as well as pathways that drive immune evasion and reduce immune cell infiltration [50,51].

**Figure 2:**
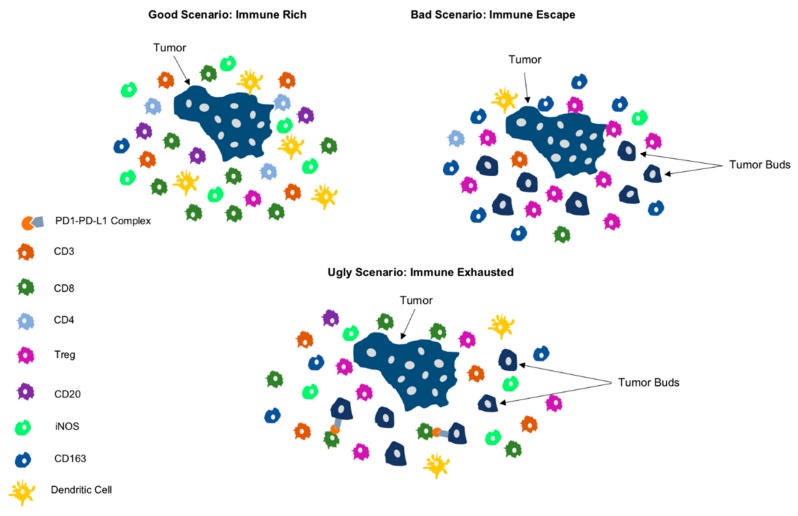
Schematic representation depicting the three main immunophenotypes of pancreatic cancer. The immune rich phenotype displays marked infiltration through cytotoxic T-cell populations, including CD3+, CD8+ and CD4+ cells, while the immune escape phenotype shows increased Foxp3+ Tregs and macrophages with M2 polarization (CD163+), along with many tumor buds. The immune exhausted phenotype shows upregulation of PD-L1 and an inflamed microenvironment with presemce of many tumor buds.

The “ugly” clinical scenario encompasses those PDACs in which the selective pressure of the highly cytotoxic immune microenvironment leads to the development of specific immune evasion mechanisms like the upregulation of the immune checkpoint molecule PD-L1 [52]. These PDACs feature an *"immune-exhausted"* immunophenotype with exceptional characteristics linking unfavorable clinicopathologic features, like high-grade tumor budding, with an unusually immunogenic, “hot” microenvironment [39]. This demonstrates that, the anti-tumor effect of the cytotoxic immune response can be reversed by the PD-L1 upregulation, rendering the microenvironment compliant with the formation of tumor buds and conferring a poor prognosis [39,52]. Graphic representation of the clinical scenarios is depicted in Figure 2.

## 6. Emerging Opportunities and Challenges

**Figure 3:**
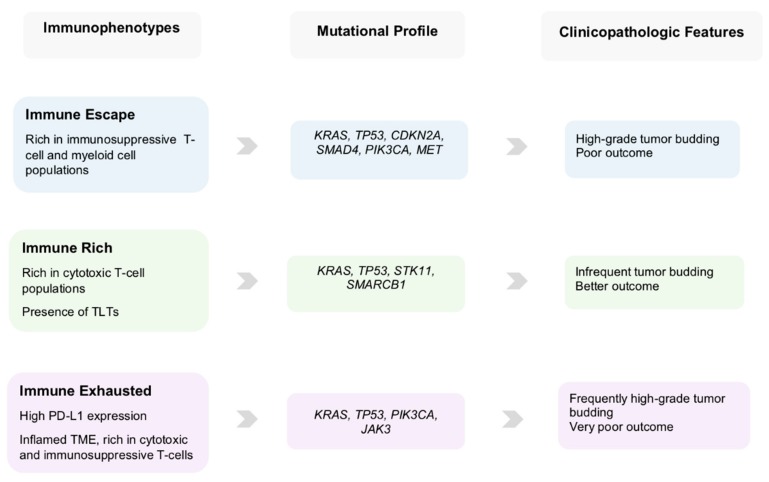
Table summarizing the immune, genetic and clinicomorphologic findings of the three immunophenotypes of pancreatic cancer.

In the present review we undertake a categorization of the PDAC TME patterns by combining genomic, immunophenotypical and clinicopathologic factors leading to distinct clinical scenarios (Figure 2 and Figure 3) with prognostic and possibly predictive value.

Recent findings from genome sequencing studies point out that PDAC is lacking highly actionable somatic mutations [10,12,13]. However, as extensive molecular profiling is becoming more and more part of the routine clinical investigation even a small number of targetable alterations may lead to beneficial therapeutic options for individual patients. For example, therapies that target the MET pathway may be effective in PDACs with presence of *MET* mutations [53]. Furthermore, germline or somatic mutations in DNA damage repair genes (*ATM, BRCA1, BRCA2*, and *PALB2)*, may sensitize these tumors to platinum-based chemotherapy or poly-(ADP-ribose) polymerase (PARP) inhibition [54].

Additionally, the ability of some tumors to develop characteristics that help them to cope with cytotoxic immune infiltrates, like the activation of the immune checkpoint molecules PD-L1 and CTLA-4, create new treatment opportunities. Although single-agent immunotherapy with checkpoint inhibitors has so far not been successful in PDAC [4], combinatorial treatments may increase therapeutic impact. For example, it has been reported that CD40 antibodies, especially in combination with chemotherapy, checkpoint inhibitory antibodies, and other immune modulators can augment T cell-dependent anti-tumor activity [55]. Despite the fact that most trials administering immunotherapy to PDAC patients are using combinatorial schemes with chemotherapy, there is currently lack of information regarding the mechanisms by which chemotherapy can promote immunogenicity and antitumor activity within the TME of PDAC, thus increasing the efficacy of immunotherapy. A recent phase II study administering gemcitabine plus nab-paclitaxel in combination with indoximod showed that responders to treatment displayed increased CD8+ T-cell density in on-treatment tumor biopsy samples [56]. Moreover, all patients, both responders and non-responders, showed increases in the CD8+ effector to Foxp3+ Treg ratio in on-treatment biopsy samples as compared with baseline [56]. These findings suggest that therapy may alter the immune microenvironment of PDAC, shifting a “cold” TME towards a “hot” one, increasing therapeutic efficacy. Therefore, information regarding the composition of the immune infiltrates in the TME of treatment naïve PDAC patients at baseline, by using the three above mentioned categories, may inform therapy decisions improving patient stratification.

Overall, based on all the above-mentioned evidence, it can be stated that the use of combinatorial immunophenotypic, genetic and morphologic data for characterizing subsets of pancreatic cancer, may lead to better association between histomorphologic findings and biological processes enabling the development of new, more individualized therapeutic strategies to improve outcomes of PDAC patients.

## 7. Conclusions

The categorization of PDAC by combining information from genomic, immunophetypical and morphological profiling will lead to a better association between histomorphological findings and biological processes. This may facilitate the development of prognostic and/or predictive biomarkers and improve patient stratification, eventually guiding individually tailored monotherapies or combinatorial treatments towards more precise therapeutic approaches for pancreatic cancer.
